# Divergent topological networks in Alzheimer’s disease: a diffusion kurtosis imaging analysis

**DOI:** 10.1186/s40035-018-0115-y

**Published:** 2018-04-27

**Authors:** Jia-Xing Cheng, Hong-Ying Zhang, Zheng-Kun Peng, Yao Xu, Hui Tang, Jing-Tao Wu, Jun Xu

**Affiliations:** 1 Department of Neurology, Northern Jiangsu People’s Hospital, Yangzhou University, Yangzhou, 225001 China; 2 Department of Radiology, Northern Jiangsu People’s Hospital, Yangzhou University, Yangzhou, 225001 China; 3 Medical Experimental Center, Northern Jiangsu People’s Hospital, Yangzhou University, Yangzhou, 225001 China; 4 Department of Neurology, Beijing TianTan Hospital, Capital Medical University, Beijing, 100050 China; 5 Jiangsu Key Laboratory of Integrated Traditional Chinese and Western Medicine for Prevention and Treatment of Senile Diseases, School of Medicine, Yangzhou University, Yangzhou, 225001 Jiangsu China

**Keywords:** Small world, Alzheimer’s disease, Diffusion kurtosis imaging, Brain networks

## Abstract

**Background:**

Brain consists of plenty of complicated cytoarchitecture. Gaussian-model based diffusion tensor imaging (DTI) is far from satisfactory interpretation of the structural complexity. Diffusion kurtosis imaging (DKI) is a tool to determine brain non-Gaussian diffusion properties. We investigated the network properties of DKI parameters in the whole brain using graph theory and further detected the alterations of the DKI networks in Alzheimer’s disease (AD).

**Methods:**

Magnetic resonance DKI scanning was performed on 21 AD patients and 19 controls. Brain networks were constructed by the correlation matrices of 90 regions and analyzed through graph theoretical approaches.

**Results:**

We found small world characteristics of DKI networks not only in the normal subjects but also in the AD patients; Grey matter networks of AD patients tended to be a less optimized network. Moreover, the divergent small world network features were shown in the AD white matter networks, which demonstrated increased shortest paths and decreased global efficiency with fiber tractography but decreased shortest paths and increased global efficiency with other DKI metrics. In addition, AD patients showed reduced nodal centrality predominantly in the default mode network areas. Finally, the DKI networks were more closely associated with cognitive impairment than the DTI networks.

**Conclusions:**

Our results suggest that DKI might be superior to DTI and could serve as a novel approach to understand the pathogenic mechanisms in neurodegenerative diseases.

## Introduction

Alzheimer’s disease (AD) is the most frequent neurodegenerative disorder causing cognitive impairment, disabilities and finally death in aged people. The upgraded guidelines recommend that the diagnosis of AD should be based on the psychiatric and neurological signs, imaging findings, and the presence of biomarkers [1]. Accumulated amyloid β peptides (Aβ) and phosphorylated tau have been speculated to play an early role in the brains affected by AD [2], followed by synaptic dysfunction, brain hypometabolism and eventually brain atrophy as the biomarkers of neurodegeneration [3–5]. Combined Tau-Aβ interactions could promote the disruption of axonal connectivity [6]. Different imaging modalities have revealed structural and functional connectivity disruptions between anatomically distinct brain regions in patients with AD. Graph theory speculates that the human brain is constructed of complex networks with topology, small world and centrality based on the measurement of structural data, diffusion tensor data and functional magnetic resonance imaging (fMRI) data [7–11]. Thus, AD has been proposed as a disconnection syndrome based on the converging findings.

Graph theoretical methods provide a powerful approach to quantitative analysis of the organization of network connectivity [7–9, 12–18]. A number of characteristic properties have been used to describe the network including characteristic path length (Lp), clustering coefficient (Cp), global efficiency (Eg) and the existing hubs. The nodes of a network are represented by brain regions or voxels defined by a predetermined parcellation scheme, while the edges depend on different modalities of imaging technology [10]. Structural networks are based on brain anatomical features such as the grey matter (GM) volume, cortical thickness, surface area, and the correlations between different regional features [7, 19, 20]. Diffusion networks can be calculated by the metric of fiber number (FN), density, strength, probability, and mean diffusion measures from the data of diffusion tensor imaging (DTI) [9, 21–23].

A recent neuroimaging study has shown that small world aberrations in structural networks in AD patients were associated with Aβ deposition [24]. Using graph theory analysis of the GM structural networks, both He et al. [7] and Yao et al. [25] revealed increased Cp and Lp of whole brain and reduced nodal centrality predominantly in the temporal-parietal association cortex regions in AD patients. However, Tijms et al. [17] reported an opposite finding about Cp and Lp in the cortical structural networks in AD. Previous studies also observed that white matter (WM) structural networks in both AD and healthy groups had a small world topology with DTI [21, 26, 27]. In addition, even in the preclinical AD, the DTI networks were found to be impaired earlier than other structural imaging markers. Furthermore, studies showed that performance in memory and executive function in AD patients were related to decreased local efficiency (Eloc), increased Lp and decreased Eg with DTI networks [9, 23, 28].

However, DTI yields only a fraction of the information potentially accessible by diffusion MRI, mainly due to the fact that the DTI is unable to quantify non-Gaussian diffusion [29]. In the brain, non-Gaussian diffusion is known to be substantial and arises from diffusion barriers, such as cell membranes and organelles as well as water-containing compartments (both extracellular and intracellular) with differing diffusion properties. In recent years, a measure of diffusional non-Gaussianity called diffusion kurtosis imaging (DKI) was developed rapidly, by which it could overcome the limitations of DTI and provide a way to investigate the microstructure of both WM and GM [30]. At least theoretically, GM tissue is of non-Gaussian distribution. The DKI model is composed of diffusion and kurtosis measurements. The kurtosis indices of mean kurtosis (MK), axial kurtosis (AK), radial kurtosis (RK) respectively represent the average apparent kurtosis along all diffusion encoding directions, the kurtosis along the directions parallel and perpendicular to the principal diffusion direction [31, 32].

DKI has been initially applied to the study of the nervous system, such as normal brain tissue, cerebral infarction, brain tumors, brain trauma as well as Alzheimer’s disease and Parkinson’s disease [33–35]. For example, DKI can efficiently assess the glioma grade and cellular proliferation [36]. Up to date, few studies have applied DKI to AD patients and only revealed decreased regional kurtosis values in both the GM and WM of the parietal and occipital lobes by regions of interest measurement, and suggested that DKI could be sensitive in the assessment of microstructure damage in AD patients [37, 38]. Another study indicated that the combination of diffusion and kurtosis measurements from DKI significantly yielded high performance in the pathological automatic detection of Alzheimer’s disease [39]. These studies, however, have not addressed the question of whether heterogeneous DKI properties are coordinated between different cortices. More specifically, human brain WM networks can be constructed using DTI metrics for a long time and present small world, centrality and modularity. Therefore, we focus on the construction of diffusion kurtosis networks (DKN) to investigate potential aberrant mechanisms underlying brain dysfunction in AD. We hypothesize that DKN could present small world properties in the human brain, and we further investigate whether the alterations of non-Gaussian diffusion properties are sensitive to the neurodegenerative disorders such as AD.

## Materials and methods

### Subjects

The subjects were recruited from Northern Jiangsu People’s Hospital, including twenty-one AD patients and nineteen normal controls (NC). The diagnosis for probably AD made by two experienced neurologists was based on the criteria of the National Institute of Neurological and Communicative Diseases and Stroke/Alzheimer’s Disease and Related Disorders Association (NINCDS/ADRDA) [40]. Exclusion criteria were any medical, neurological, or psychiatric conditions that could account for the symptoms of dementia, and Fazekas scale of white matter hyperintensities>2 on brain MRI. The control group was recruited according to the distribution of the age, gender and education years in the AD group. All the participants were assessed referring to a standard clinical protocol, which involved the inquiry of the medical history, an interview with a spouse or close family member, blood tests, MRI of the brain according to a standard protocol and a set of neuropsychological assessments including Mini Mental State Examination (MMSE) and Montreal cognitive assessment (MoCA).

The study was approved by the Ethics Committee of Northern Jiangsu People’s Hospital, Yangzhou University, and all participants or their guardians signed informed consent forms before MRI scanning.

Images were acquired in a 3.0 T GE Discovery MR750 scanner (GE Healthcare Systems, Milwaukee, WI, USA) system with an 8-channel head coil. DKI images were acquired with 3 b-values (b = 0, 1250 and 2500 s/mm^2^) along 30 diffusion gradient directions using a single-shot EPI sequence. DKI sequence parameters were: TR = 5800 ms, TE = 100 ms; FOV = 240 × 240 mm^2^; matrix = 100 × 100; voxel = 2.4 × 2.4× 4 mm^3^; 35 axial slices. 3D T1WI images of the whole brain were acquired using the BRAVO sequence, the imaging parameters were: TR = 8.2 ms; TE = 3.2 ms; TI = 450 ms; flip angle = 12^°^; FOV = 256 mm × 256 mm; matrix = 256 × 256; voxel = 1 × 1 × 1 mm^3^; 160 slices.

### DKI data processing

DKI data were processed by using Matlab R1202b and Diffusion Kurtosis Estimator (DKE) (http://www.nitrc.org/projects/dke) [41]. Data were first processed to correct subject motion, eddy current-induced geometric distortions and denoise. The DKI model was parameterized by the diffusion tensor (DT) and kurtosis tensor (KT) from which several rotationally-invariant scalar measures were extracted, which could be used to construct WM sensitive and GM sensitive networks respectively. The CLLS-QP algorithm used in DKE for extracting DT and KT parameters has been described in detail in the previous research [41]. The DT-derived measures included mean diffusivity (MD), axial diffusivity (Ad), radial diffusivity (RD), fractional anisotropy (FA) [42]; and KT-derived measures were AK, RK, MK and kurtosis fractional anisotropy (KFA) [41, 43]. Of the DT-derived measures, the MD corresponds to the diffusion coefficient averaged over all possible diffusion directions, whereas Ad is the diffusion coefficient in the direction of the principal diffusion tensor eigenvector and RD is the diffusion coefficient averaged over all diffusion directions perpendicular to the principal diffusion tensor eigenvector. FA measures the degree of anisotropy and ranging between 0 (fully isotropic diffusion) and 1 (fully anisotropic diffusion) [44]. The data were used in fiber tracking (FT) module embedded in DKE to get the outputs for further use.

### Cortical parcellation

Each individual high-resolution structural image (T1 image) was first coregistered to the FA image in the diffusion space using a linear transformation. The transformed structural image was then mapped to the T1 template of the Montreal Neurological Institute (MNI) space using a nonlinear transformation [45]. The resulting inverse transformation was then used to warp the Automated Anatomical Labeling (AAL) mask from the MNI space to the individual native space.

### Network construction

GM network was defined as a correlation network based on each DKI parameter measurement. A regional parameter was computed as the average value of all voxels within the region. The interregional correlation matrix of each group was then obtained by calculating the correlation coefficients across individuals between the regional parameters of every pair of regions using Pearson’s correlation. Therefore, AD and control groups achieved a parameter matrix for each DKI metric respectively. The grey matter network contained N nodes and K edges, which included 90 cortical and sub-cortical regions in the AAL atlas (45 for each hemisphere) [46], the parameter correlation value between the two regions across all subjects meant the K values.

To construct DKI WM network, the brain nodes were also defined by the AAL atlas. Each edge represented the connecting fiber that linked a couple of brain nodes. The weights of the edges were defined as FN, mean fiber length and mean diffusion measures, including FA, MD, Ad, RD, MK, RK, AK and KFA. All the image processes of kurtosis diffusion network construction were manipulated using PANDA (http://www.nitrc.org/projects/panda) [47].

Each correlation matrix was thresholded over a wide range of sparsity (6% -40%), and the properties of the resulting graphs at each threshold value were estimated. Subsequent indicators were calculated in the Gretna (http://www.nitrc.org/projects/gretna/) [48].

### Small world analysis

Small world measures of a network (Cp and Lp) were originally proposed by Watts and Strogatz [49]. For both KT and DT networks, we calculated the Cp (the number of existing connections among the neighbors of the node divided by all their possible connections), Lp (the average minimum number of connections that link any two nodes of the network), and betweenness centrality (the number of the shortest paths between any two nodes that run through a node). A real network was defined as small world if it met the following criteria: γ = Cp/mean(Cprand) > 1 and, λ = Lp/mean(Lprand) ≈ 1 [49], where the Cprand and Lprand were the mean Cp and Lp of matched random networks that preserve the same number of nodes and edges as the real network [50, 51].

### Statistical analysis

The significance threshold was set top ≤0.05 for all analyses. All continuous variables were tested for normal distribution within groups using the Kolmogorov–Smirnov test. Differences between AD subjects and NC regarding demographical data (age, sex), MMSE and MoCA scores were calculated as follows: the chi-square test was used for categorical variables; the t-test was used for continuous normally distributed variables.

To determine the differences in GM graph network parameters between groups, a nonparametric permutation test method was used [52]. First, Cp and Lp of the networks at a given sparsity were computed separately for the AD and control groups. To test the null hypothesis that the observed group differences could occur by chance, we then randomly reallocated each subject’s set of regional mean kurtosis measures to one or the other of the two groups and recomputed the correlation matrix for each randomized group. We then obtained corresponding weighted matrix using the same sparsity threshold as in the real brain networks. Next, we calculated the network parameters for each randomized group and obtained their differences between the randomized groups. This randomization procedure was repeated 1000 times.

To determine the between-group differences in the small world properties and network efficiency of the WM networks, an analysis of covariance (ANCOVA) was performed on each diffusion metric. Age and gender were taken as covariates in this model. The relationship between the network metrics and MMSE and MoCA scores in the patient group was analyzed by the partial correlation analysis.

## Results

### Demographics

There were no significant differences in both age (*p* = 0.07) and gender (*p* = 0.8) of AD patients and controls. For the neuropsychological tests, there were significant differences in MMSE and MoCA scores between the two groups (p<0.05, Table 1).

**Table 1:** Demographic and clinical characteristics of subjects

Characteristics	AD Patients	Controls	*P* value
Age	73.57(±6.87)	70 (±8.08)	0.07
Female/male	12/21	9/18	0.8
MMSE	18.71 (±5.9)	28.16 (±1.12)	<0.001
MoCA	15(±6.67)	27.89(±0.14)	<0.001

Scores are shown with mean (±SD)

### Within group network analysis

#### Small world analysis of the grey matter networks

**Fig. 1:**
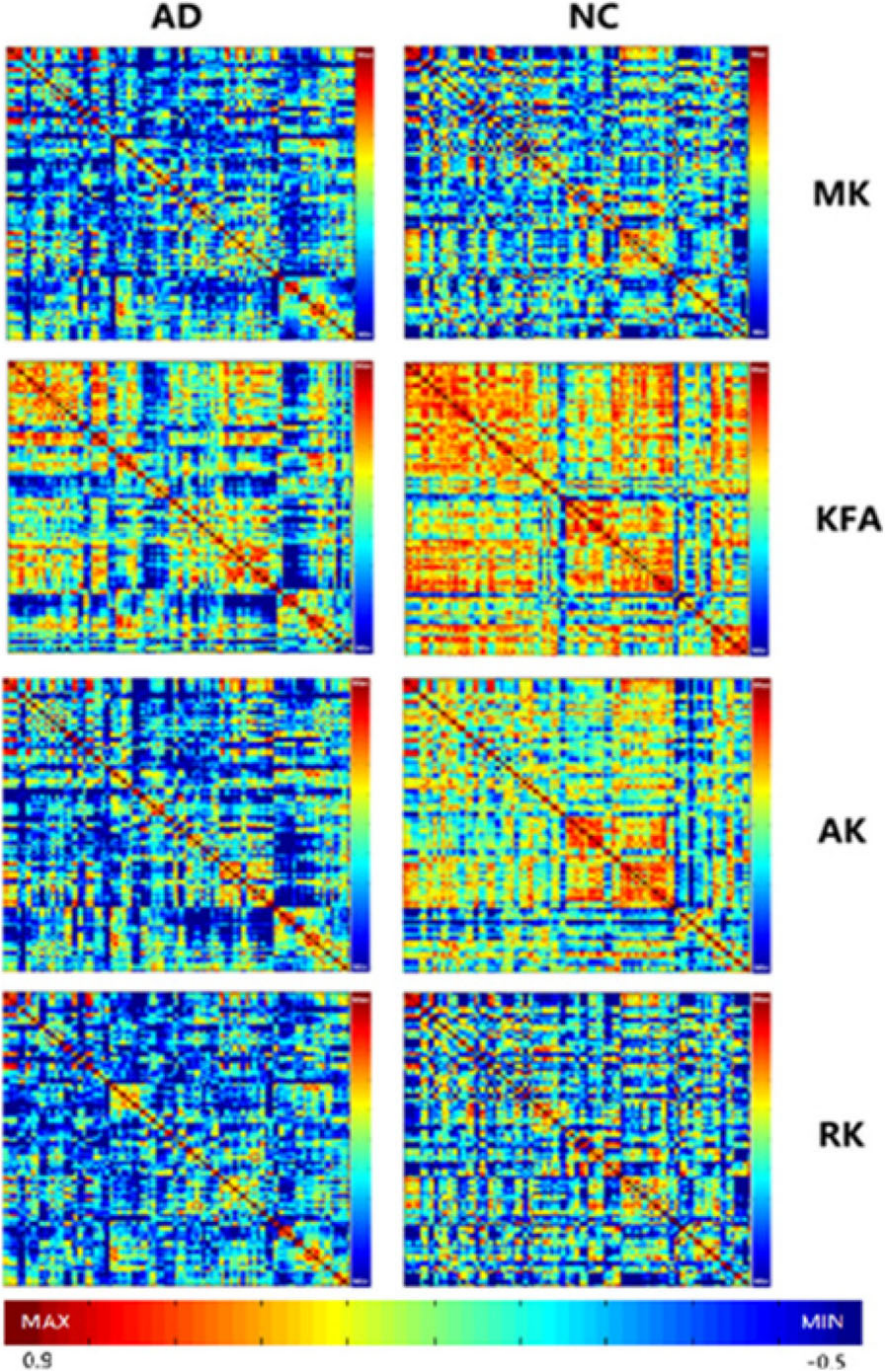
The interregional correlation matrix (90 × 90) in the AD and NC groups using DKI metrics of MK, KFA, AK and RK. The color bar indicates the value of the interregional parameter correlation. The red color bar represents the higher positive correlation value. The blue color bar represents the higher negative correlation value. From the maps, a great degree of dispersion in DKI could be observed in AD patients. Note more strong positive coordinated effects existing in extensive brain regions labeled by red color for the metric of MK, KFA, AK and RK in the control group vs. AD group, and KFA is the typical. The higher KFA value meant more compact histological structure

The interregional parameter correlation values of the cortical networks were calculated to construct correlation matrices (90 × 90) for the NC and AD groups. The images of the group level interregional correlation matrices using DKI metrics of MK, KFA, AK and RK are shown in Fig. 1, and KFA in the control group presented the strongest positive coordinated effects during observations among these metrics.

**Fig. 2:**
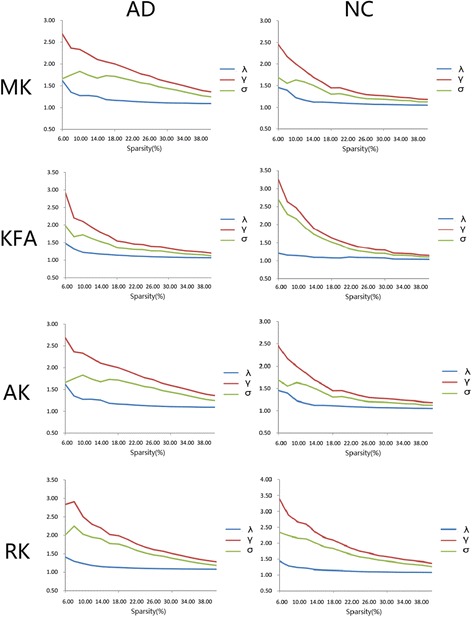
Small world properties of GM networks in the AD and NC using DKI metrics of MK, KFA, AK and RK. Both networks demonstrated small world architectures over a wide range of sparsity (6% ~ S ~ 40%) in comparison with the matched random networks. γ value was calculated by Cp/mean (Cprand), λ value was calculated by Lp/mean (Lprand). The small-worldness value of σ was presented by γ/λ

We found the small world attributes of GM networks with MK, KFA, AK and RK metric in the normal elder subjects and AD patients over a wide range of sparsity (6% ~ S ~ 40%) (showed in Fig. 2). The small-worldness values (σ = γ/λ) calculated from the DKI indices were larger than 1, the values with MK, KFA, AK and RK were 1.57, 1.67, 1.75 and 2.25 in AD patients, respectively, and were 2.88, 2.28, 1.55, 2.24 in controls, respectively.

#### Small world analysis of the white matter networks

As expected, we also observed that the brains of both the AD patients and controls had prominent small world network properties in WM networks over a wide range of sparsity (6% ~ S ~ 40%) in comparison with the matched random networks. The small-worldness values calculated from all DKI indices were larger than 1, the values with FA, MD, Ad, RD, MK, RK, AK and KFA were 1.57, 1.50, 1.28, 1.54, 2.04, 1.65, 2.96, 1.70 in AD patients, respectively, and were 1.32, 1.57, 1.32, 1.76, 1.47, 1.33, 1.72, 1.43 in controls, respectively (Table 2). The small-worldness values constructed by FN were 3.02 in AD patients and 1.84 in controls.

**Table 2:** Comparison of DKI metrics networks between control and AD group

				KT				DT			
		FN	Length	MK	KFA	AK	RK	MD	FA	Ad	RD
Lp	AD	0.12(0.02)*	0.02(0.01)*	2.53(0.12)*	6.64(0.39)*	4.47(0.36)*	2.8(0.23)*	2.58(0.25)*	7.35(0.36)*	1.78(0.09)*	2.25(0.6)*
	NC	0.03(0.01)	0.05(0.01)	4.44(1.16)	9.63(2.69)	6.25(1.28)	4.32(0.93)	4.06(0.91)	12.54(3.06)	3.12(0.68)	4.72(1.05)
Cp	AD	0.03(0.01)*	0.76(0.01)*	0.26(0.02)*	0.17(0.03)*	0.25(0.03)	0.13(0.02)*	0.11(0.01)*	0.14(0.02)*	0.1(0.01)*	0.11(0.02)
	NC	0.12(0.03)	0.11(0.02)	0.3(0.04)	0.26(0.38)	0.24(0.02)	0.18(0.03)	0.13(0.02)	0.19(0.02)	0.13(0.02)	0.12(0.02)
λ	AD	1.08(0.04)	1.04(0.02)	1.06(0.02)*	1.05(0.02)	1.15(0.4)*	1.06(0.02)*	1.11(0.05)*	1.05(0.01)*	1.06(0.03) *	1.07(0.04)*
	NC	1.12(0.05)	1.06(0.03)	1.03(0.01)	1.04(0.14)	1.04(0.01)	1.03(0.02)	1.04(0.02)	1.03(0.01)	1.04(0.02)	1.04(0.02)
γ	AD	3.27(0.69)*	1.55(0.3)*	2.16(0.21)*	1.47(0.26)	3.41(0.49)*	1.75(0.39)*	1.67(0.24)	1.64(0.32)*	1.35(0.19)	1.66(0.4)
	NC	2.06(0.38)	1.39(0.16)	1.49(0.19)	1.49(0.16)	1.79(0.22)	1.36(0.18)	1.63(0.27)	1.36(0.12)	1.38(0.16)	1.84(0.29)
σ	AD	3.02(0.57)*	1.48(0.26)*	2.04(0.19)*	1.7(0.3)*	2.96(0.4)*	1.65(0.34)*	1.5(0.25)	1.57(0.29)*	1.28(0.19)	1.54(0.34)
	NC	1.84(0.36)	1.31(0.15)	1.47(0.13)	1.43(0.14)	1.72(0.2)	1.33(0.16)	1.57(0.25)	1.32(0.11)	1.32(0.13)	1.76(0.27)
Eloc	AD	9.52(1.97)*	21.78(4.15)*	0.47(0.03)	0.11(0.02)*	0.34(0.03)	0.48(0.04)*	0.50(0.48)	0.17(0.01)*	0.42(0.05)*	0.44(0.05)*
	NC	25.40(5.46)	31.90(3.58)	0.45(0.06)	0.19(0.03)	0.36(0.47)	0.54(0.06)	0.50(0.52)	0.17(0.02)	0.57(0.06)	0.42(0.05)
Eg	AD	8.38(1.17)	34.53(2.63)*	0.4(0.23)*	0.13(0.01)	0.23(0.02)*	0.36(0.03)*	0.39(0.35)*	0.15(0.07)*	0.56(0.03)*	0.47(0.12)*
	NC	8.67(1.84)	19.72(4.53)	0.24(0.05)	0.11(0.26)	0.16(0.28)	0.24(0.05)	0.26(0.47)	0.09(0.02)	0.33(0.06)	0.22(0.04)

*p< 0.05, corrected. γ value was calculated by Cp/mean(Cprand), λ value was calculated by Lp/mean(Lprand). The small-worldness value of σ was presented by γ/λ. Scores are shown with mean (±SD)

#### Hub region analysis of the grey matter networks

**Fig. 3:**
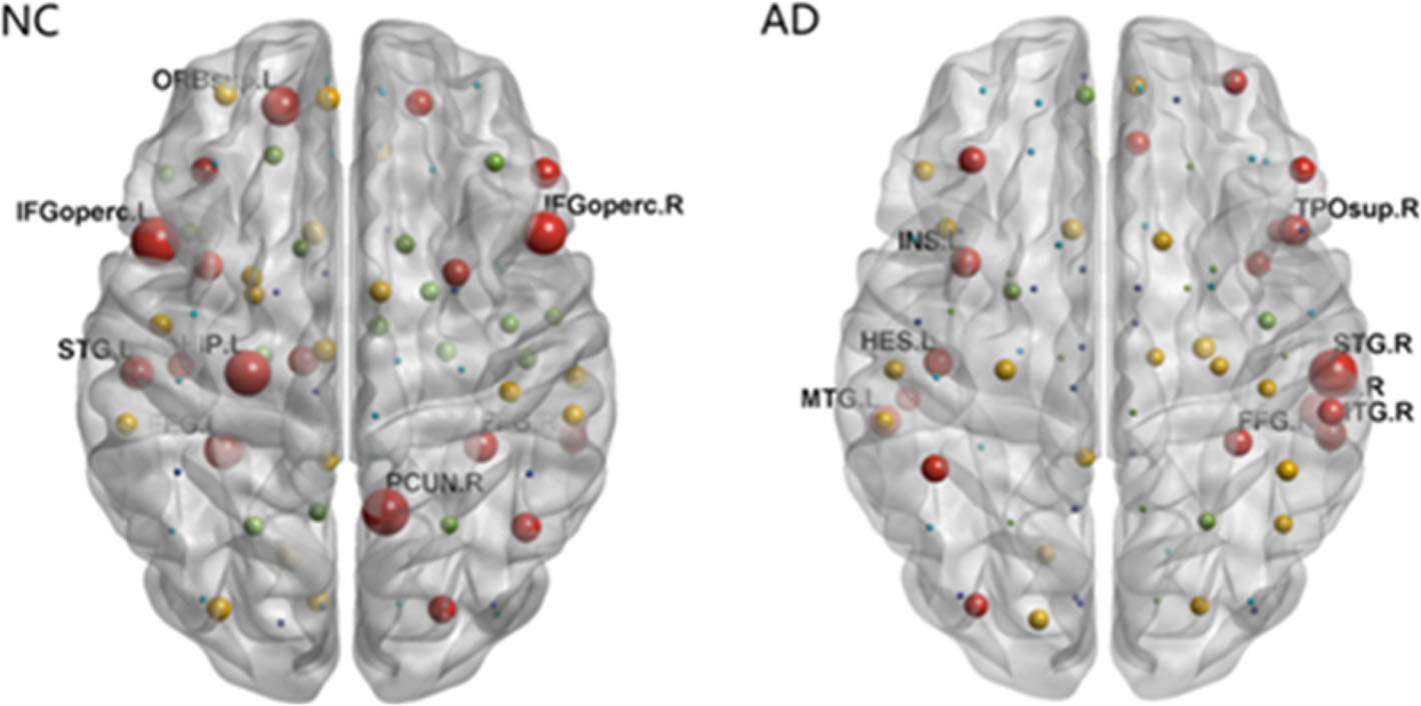
Hub regions within groups for MK metric networks. Each ball represented corresponding brain region in AAL atlas, displayed in the center of the region. The size of balls represented the bi value. Only the hub regions with bi>1.5 were indicated by red. Note the hub regions in AD became sparse in the default mode areas. The figure was processed by BrainNet Viewer software. IFGoperc.L: Left inferior frontal gyrus, opercular part; PCUN.R: Right precuneus; IFGoperc.R: Right inferior frontal gyrus, opercular part; STG.L: Left superior temporal gyrus; STG.R: Right superior temporal gyrus; FFG.L: Left fusiform; FFG.R: Right fusiform; ORB.sup.L: Left superior frontal gyrus, medial orbital; HIP.L: Left hippocampus; MTG.L: Left middle temporal gyrus; MTG.R: Right middle temporal gyrus; TPOsup.R: Right temporal pole, superior temporal gyrus; INS.L: Left insula; HES.L: Left heschlgyrus

To identify the hub regions, we examined normalized nodal betweenness centrality for the MK metric (bi, bi = Bi/B, where B was the average betweenness of the network. bi was a global centrality measure that captured the influence of a node over information flow between other nodes in the network) of each cortical region in both groups. When the betweenness value of a node was more than 1.5 times the average betweenness of the network (bi>1.5), the node was considered as a hub [53]. In the control group, 10 regions including 4 association cortex regions and 6 paralimbic cortex regions were identified as the hubs (Table 3), and in the AD group, 9 association cortex regions were identified as the hubs (Table 4). These identified hubs were predominately located in regions of association cortex such as superior temporal gyrus, middle temporal gyrus, middle frontal gyrus and fusiform gyrus (Fig. 3), and compared to the controls, less hub regions were observed in thalamus and paralimbic system in AD patients, which were mostly involved in default mode.

**Table 3:** Regions showing high betweenness centrality in DKN of control subjects

Regions	Class	Normalized betweenness, bi
Left inferior frontal gyrus, opercular part	Paralimbic	4.69
Right precuneus	Paralimbic	4.40
Left hippocampus	Paralimbic	4.26
Right inferior frontal gyrus, opercular part	Paralimbic	4.05
Left superior frontal gyrus, orbital part	Paralimbic	3.40
Left fusiform gyrus	Association	3.30
Left superior temporal gyrus	Association	2.79
Right fusiform gyrus	Association	2.67
Left thalamus	Paralimbic	2.24
Right inferior frontal gyrus, triangular part	Association	2.20

The hub regions (bi>1.5) in the GM network of the control group for the MK metric were listed in a descending order of their normalized betweenness, bi. The regions were classified as primary, association and paralimibic as described by Mesulam (1998)

**Table 4:** Regions showing high betweenness centrality in DKN of AD patients

Regions	Class	Normalized betweenness,bi
Right superior temporal gyrus	Association	6.87
Right inferior temporal gyrus	Association	3.96
Right middle temporal gyrus	Association	3.62
Left middle temporal gyrus	Association	3.52
Right temporal pole, superior temporal gyrus	Paralimbic	3.44
Left insula	Association	3.19
Left heschl gyrus	Association	3.04
Right fusiform gyrus	Association	2.86
Left Inferior parietal, but supramarginal and angular gyri	Association	2.64
Left middle frontal gyrus	Association	2.55

The hub regions (bi>1.5) in the GM network of the AD group for the MK metric were listed in a descending order of their normalized betweenness, bi. The regions were classified as primary, association and paralimibic as described by Mesulam (1998)

### Between group network analysis

#### Comparison in grey matter networks

**Fig. 4:**
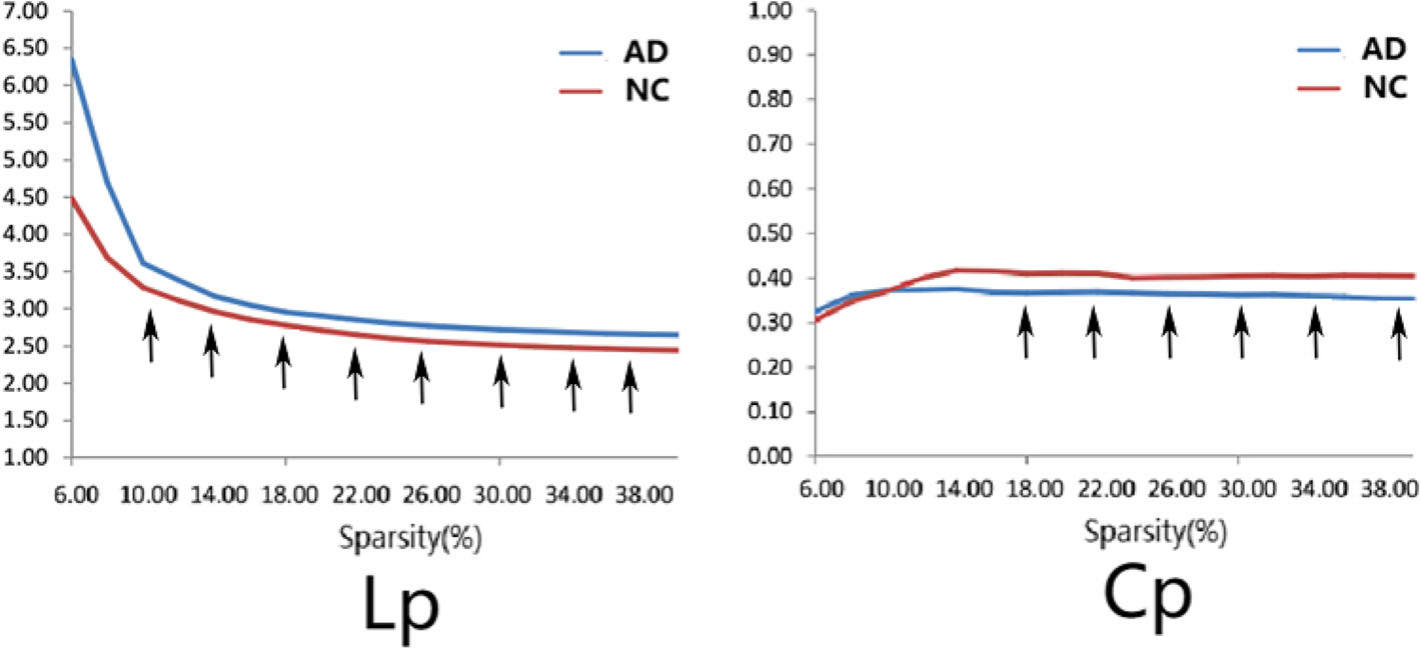
Between-group grey matter differences in Lp and Cp properties. The graph shows AD patients (blue lines) presented with increased Lp values and decreased Cp values in the brain networks than controls (red lines) over a wide range of thresholds. Statistical analysis of the between-group differences obtained by 1000 permutation tests further revealed significant differences (p< 0.05) in the Lp values at 10%<S<40% and Cp values at 14%<S<40% (the black arrows)

For the MK metric of DKI networks over a wide range of sparsity, AD patients showed increased Lp and decreased Cp compared with controls (showed in Fig. 4). Permutation test further revealed significant differences (p< 0.05) in the Lp values at 10%<S<40% and Cp values at 14%<S<40%.

#### Comparison in white matter networks

**Fig. 5:**
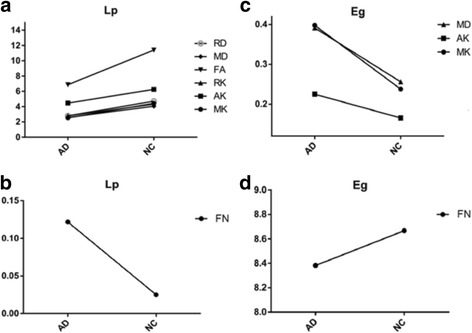
Comparison of small world properties in the WM networks between AD and NC group. **a**. Lp values of DKI metrics; **b**. Lp values of FN; **c**. Eg of DKI metrics; **d**. Ad: Eg of FN. Divergence network features in Lp and Eg were displayed by various DKI parameters

We constructed WM networks by using diffusion probabilistic tractography, and compared with controls, networks of FN in AD patients showed significantly increased Lp and decreased Eg (Fig. 5). For the WM networks with the DKI metrics of MK, AK, RK, KFA, FA, MD, RD and Ad, AD patients showed significantly decreased Lp compared with controls (p<0.05, corrected). Most of the DKI metrics showed decreased Cp except AK and RD in AD (p<0.05, corrected). For efficiency measurements of the WM networks, the Eloc and Eg were computed. Compared with the controls, no significant alterations in the Eloc were observed in AD patients, whereas significantly increased Eg was found using MK, AK and MD metrics. The differences in network metrics were listed in Table 2. These findings indicated that distinct small world network properties could be derived from different DKI indices in AD patients.

#### Comparison of small word attributions between KT and DT networks

As shown in Table 2, KT and DT small world attributes (Lp, Cp, λ, γ and σ) in AD were listed. We found that AD patients had increased σ value for all KT-derived indices and merely FA of DT-derived indices. Similarly, MK and RK from KT-derived indices showed significant differences concurrently in the small world attributes of Lp, Cp, λ and γ, in contrast to only FA from DT-derived indices.

#### Relationship of small world networks and cognitive performance

We next examined the relationships between the network metrics (small world and efficiency metrics) and cognitive performance. Partial correlation analysis with age and gender as confounding covariates were separately performed for the AD group. The results showed that KT metrics had closer ties with cognitive functions than DT metrics, particularly for FN, MK, KFA and AK metrics (p<0.05, corrected. See Table 5). We found that MMSE scores were significantly positively correlated with Lp of MK, λ of RK as well as Eloc of FN, KFA and RK, while negatively correlated with σ of FN, MK and KFA. In addition, MoCA scores were significantly positively correlated with Lp of AK, λ of fiber length, RK, MD, as well as Eloc of FN, RD and AK, while negatively correlated with σ of FN, MK and Eg of AK.

**Table 5:** Partial correlation analysis between small world DKI networks and cognitive performances in AD patients

				KT				DT			
		FN	Length	MK	KFA	AK	RK	MD	FA	Ad	RD
Lp	MMSE	0.01	-0.18	0.33*	0.20	0.50	0.46	-0.06	0.06	-0.04	-0.07
	MOCA	0.20	-0.09	0.25	-0.07	0.58*	0.41	-0.13	-0.09	-0.07	-0.03
Cp	MMSE	0.16	-0.75	-0.11	0.11	-0.03	-0.08	0.37	0.20	-0.14	0.11
	MOCA	0.11	-0.70	-0.06	0.20	-0.03*	-0.12	0.40	0.21	-0.16	0.13
λ	MMSE	-0.42	0.77	-0.28	-0.43	0.22	0.48*	0.08	-0.06*	-0.40	0.24
	MOCA	-0.43	0.70*	-0.25	0.30	0.29	0.41*	0.09*	-0.15	-0.53	0.27
γ	MMSE	0.43*	-0.76	0.19*	0.45*	-0.20	-0.46	-0.09	0.07	0.41	-0.26
	MOCA	0.45*	-0.70	0.36	-0.08	-0.27	-0.39	-0.10	0.19	0.55	-0.29
σ	MMSE	-0.44*	0.73	-0.19*	-0.45*	0.21	0.46	0.09	-0.07	-0.42	0.26
	MOCA	-0.45*	0.65	-0.30*	0.08*	0.27	0.38	0.10	-0.19	-0.56	0.29
Eloc	MMSE	0.22*	0.58	0.20*	0.21*	-0.11*	-0.07	-0.30	-0.03	-0.08	-0.02
	MOCA	0.21*	0.62	0.16	-0.33	0.19*	0.08	-0.30	-0.02	-0.17	0.06*
Eg	MMSE	0.02	-0.37*	0.37	0.11	0.50*	0.47	-0.11	0.07	-0.03	-0.08
	MOCA	0.11	-0.35*	0.31	0.20	0.58*	0.42	-0.18	-0.07	-0.06	-0.05

The partial correlations were computed with age and gender as the confounding covariates. *p < 0.05, corrected. γ value was calculated by Cp/mean(Cprand), λ value was calculated by Lp/mean(Lprand). The small-worldness value of σ was presented by γ/λ. Note there are more KT metrics exhibiting significant correlations to cognitive performances than DT metrics

## Discussion

Our current study investigated DKN in AD patients and healthy controls. To our best knowledge, no previous report on the construction of DKI networks has so far been published. The main findings are as follows: (1) DKN presented small world characteristics in the brains of both healthy subjects and AD patients. (2) GM network of AD patients tended to be a less optimized network. (3) AD WM networks showed increased Lp and decreased Eg for FN, by contrast, decreased Lp and increased Eg for the other diffusion metrics of MK, RK, AK, KFA, FA, MD, RD and Ad. (4) Regional hubs decreased in AD. (5) Kurtosis parameters had stronger associations with cognitive impairment in AD patients than the DT-derived parameters.

Alterations of small world topological properties in AD have been demonstrated by using different modalities such as Electroencephalogram (EEG), Magnetoencephalogram (MEG), fMRI, structural magnetic resonance imaging (sMRI) and DTI in plenty of previous studies. However, there are inconsistent findings about the small world properties. Most structural connectivity networks exhibited increased Lp in AD-related networks. For example, by sMRI and DTI network analysis, Cp and Lp were showed significantly increased in AD patients compared to the controls [7, 9]. On the contrary, decreased Lp in AD was also demonstrated by using sMRI and DTI [17, 54]. For functional connectivity network analysis, EEG and fMRI studies showed that AD patients had increased Lp [8, 55]. In contrast, decreased Lp was found in brain functional networks of AD patients using fMRI [56], EEG [57] and MEG [58]. The discrepancies in the brain network analysis could be due to different modalities. Our findings with DKI networks could integrate the discrepancies.

### Divergent features in WM network metrics

The connectivity weights of the white matter networks were defined with the DT and KT metrics that represented distinct biological significance. The weighted networks retain the biophysical information in the graph model. MK reflects the general tissue complexity and the lesion heterogeneity [59]. AK is mainly affected by the cellular structure, and RK is mainly affected by the cell membrane and the myelin sheath [60]. KFA is mathematically analogous to FA, and provides complementary information about anisotropy in diffusion dynamics to describe deep brain structures [61]. The higher KFA value means more compact histological structure. In our findings of KFA matrixes, reduced coordinated effects between the extensive brain regions in AD meant loosened microstructure. The Ad might assess the integrity of axonal conduction. RD might be an adequate parameter for the assessment of myelin integrity [62].

In our findings, divergent features in WM network alterations presented in AD brains with increased Lp and decreased Eg for FN, on the contrary, for most of the diffusion metrics such as MK, RK, AK, KFA, FA, MD, RD and Ad with decreased Lp and increased Eg findings. In addition, most of the DKI metrics showed significant decreases in Cp in the AD group. Increased Lp implied the diminished information propagation ability of the fiber tract, and similar findings were demonstrated in previous studies using DTI and fMRI [8, 9, 63, 64]. For the observation of decreased Lp and increased Eg, we speculated that it could result from progressive dedifferentiation among different domain-specific cortical regions in AD brains. This kind of dedifferentiation tendency has been revealed in normal aging people and AD patients. For example, age-dependent epigenetic assimilation was correlated with increased similarity between the cerebral cortex and the cerebellum, indicating potential brain cell dedifferentiation [65]. Different networks were also observed to merge with aging by fMRI measurements from a macro perspective [66].

We assumed that the index of FN and the other sides of DKI reflected different aspects of the brain. DKI could quantify the deviation of water molecule diffusion from the Gaussian distribution in contrast to DTI Gaussian model. All the DKI metrics reflect microstructure complexity from various diffusion models, and when damaged, for example due to deposition of amyloid plaques and tangles, the well-organized and complex microstructures changed as well as the relevant kurtosis value [39]. However, this abnormally changing direction was not determinate, leading to divergent effects as our findings showed.

### Disruption of grey matter networks

In contrast to most diffusion MRI studies that focus merely on white matter networks, our study paid attention to changes in both GM and WM networks. In our work, we constructed GM network based on each DKI parameter measurement and found small word properties, which is consistent with previous studies based on cortical thickness [7]. The alterations of increased Lp and decreased Cp in AD indicated a less optimized network. We assumed the damage of small-worldness in AD patients was due to the changes in the tissue microstructure, and could be caused by astrogliosis, microglial activation, vascular hyalinization and axonal loss in the pathology of AD.

### Comparison between DKI and DTI

We contrasted the KT- with DT-derived networks. Firstly, our findings revealed impairment in grey matter networks by KT-derived metrics in AD patients, which is not assessable with DT-derived indices. Secondly, for the between-group comparison in the white matter networks (Table 2), all KT-derived indices, contrast to merely FA in DT-derived indices, demonstrated sensitivity to the AD brains with significantly increased small-worldness values (σ). FA has been a commonly used metric during the construction of DTI networks, and FA was considered as a biomarker of axonal integrity in the past. What’s more, our findings provided additional diffusion indices sensitive to the degenerative brain impairment. We considered that MK and RK could be optimal choices to describe the white matter networks. Evidence in previous studies indicated that MK was sensitive to demyelination, axonal damage and loss, compared to DTI analysis [67, 68]. RK might capture the diffusion heterogeneity arising from axonal membranes and myelination, may also potentially serve as a more sensitive marker in white matter [60, 62]. Finally, the alterations of diffusion kurtosis network showed a close association with the cognitive performance in our findings. More specifically, kurtosis parameters of MK, AK and KFA had stronger correlations with the cognitive performance in AD patients than the DT-derived analogical parameters.

Our findings suggested the wide range of pathological detection of kurtosis might provide more information than diffusion tensor by determining intrinsic microstructural properties with non-Gaussian measurements.

### Hub changes of small world networks

Hub nodes play an important role in transferring information between different parts of the brain network, which determines the integration efficiency of the neural information and the stability of the network structure in the networks. Our results showed that hub regions were mostly involved in the default mode in the control group, which were consistent with the previous work [69]. In contrast, hub regions within the DKI networks diminished in the AD group. Previous studies have demonstrated that loss of connectivity in default network is a possible mechanism of AD brain damage [24, 70, 71]. In addition, we found that some hub regions supervising language were impaired, including inferior frontal operculum and superior temporal gyrus.

Our study has some limitations. At first, our sample size is limited. Secondly, the exact relationship between varied indices of DKI and the complexity of brain remains not entirely clear. The reliability and reproductivity of the DKI networks remain to be confirmed in further studies.

## Conclusions

We observed interesting small world properties with DKI in human brain and impaired small world networks in the AD pathology, and divergent WM network features were demonstrated in AD brains. In addition, the damage of DKI networks was associated with cognitive performance. DKI parameters might have the superiority to DTI and could be used as a neuroimaging marker for AD by characterizing the microstructure of WM and GM.

## Abbreviations

AD: Alzheimer’s disease
Ad: axial diffusivity
AK: axial kurtosis
Cp: clustering coefficient
DKE: Diffusion Kurtosis Estimator
DKI: diffusion kurtosis imaging
DKN: diffusion kurtosis networks
DT: diffusion tensor
DTI: diffusion tensor imaging
Eg: global efficiency
Eloc: local efficiency
FA: fractional anisotropy
FN: fiber number
FT: fiber tracking
KFA: kurtosis fractional anisotropy
KT: kurtosis tensor
Lp: characteristic path length
MD: mean diffusivity
MK: mean kurtosis
MMSE: Mini Mental State Examination
MoCA: Montreal cognitive assessment
RD: radial diffusivity
RK: radial kurtosis
sMRI: structural magnetic resonance imaging
